# Gene variants associated with obstructive sleep apnea (OSA) in relation to sudden infant death syndrome (SIDS)

**DOI:** 10.1007/s00414-020-02480-0

**Published:** 2021-02-08

**Authors:** J. Kerz, P. Schürmann, T. Rothämel, T. Dörk, M. Klintschar

**Affiliations:** 1 Institute of Legal Medicine (OE 5500), Hannover Medical School, Carl-Neuberg-Str. 1, 30625 Hannover, Germany; 2 Gynecology Research Unit, Hanover Medical School, Carl-Neuberg-Str. 1, 30625 Hannover, Germany

**Keywords:** SIDS, OSA, Genetic predisposition, Hypoxia, Heart failure, Cardiovascular system

## Abstract

**Background:**

Both obstructive sleep apnea (OSA) and (at least a fraction of) sudden infant death syndrome (SIDS) are associated with impaired respiration. For OSA, an association with several gene variants was identified. Therefore, our hypothesis is that these polymorphisms might be of relevance in SIDS as well.

**Methods:**

Twenty-four single nucleotide polymorphisms (SNPs) in 21 candidate genes connected to OSA, were genotyped in a total of 282 SIDS cases and 374 controls. Additionally, subgroups based on factors codetermining the SIDS risk (age, sex, season, and prone position) were established and compared as well.

**Results:**

Two of the analyzed SNPs showed nominally significant differences between SIDS and control groups: rs1042714 in *ADRB2* (adrenoceptor beta 2) and rs1800541 in *EDN1* (endothelin 1). In the subgroup analyses, 10 further SNPs gave significant results. Nevertheless, these associations did not survive adjustment for multiple testing.

**Conclusions:**

Our results suggest that there might be a link between SIDS and OSA and its resulting respiratory and cardiovascular problems, albeit this predisposition might be dependent on the combination with other, hitherto unknown gene variants. These findings may encourage replication studies to get a better understanding of this connection.

**Supplementary Information:**

The online version contains supplementary material available at 10.1007/s00414-020-02480-0.

## Introduction

Sudden infant death syndrome (SIDS) is the unexpected death of children who die before the end of their first year of life. Their death remains unexplained even after a complete postmortem investigation, including autopsy, examination of the scene of death, and thorough review of the case history [1]. Hence, SIDS is a diagnosis of exclusion [2]. The incidence rate of SIDS was highly reduced in the USA since the 1990s, due to the “Back to Sleep” campaign [3–5], but it is still the leading cause of death of infants in developed countries. Nowadays, there are about 0.5–2.5 cases per 1000 live births [2].

One of the main hypotheses is the triple risk model which looks at three different aspects that could all lead to SIDS if they interact with each other. In this model, all three factors have to be present for SIDS to occur. These risks include first, there is an initial predisposition that is possibly genetic. Many studies, including this one, have had a look at different genetic variations but there has not yet been a factor that would fully explain the cause of SIDS. Secondly, there is the vulnerable developmental stage of vital systems such as the central nervous system (CNS) or the immune system during the first year after birth. Thirdly, there are triggering events that increase the risks of SIDS [6]. Sleeping in a prone position [3–5] is stressful and may thus be one of these factors, as well as mild airway infections. Maternal risk factors such as smoking (prenatal and postnatal) [7], and environmental factors—for example, thermal stress (warmth) and soft bedding have also been identified to increase the risk for SIDS. These risk factors have been studied and confirmed in recent studies [8–10].

Obstructive sleep apnea (OSA), on the other hand, is a chronic disease in children (affecting 1–4% [11]) or adults, which causes shallow breathing during sleep, and short periods of apnea [12–16]. A similar behavior has been observed with infants. There is a higher incidence rate amongst men than women suffering from OSA (9 to 4% respectively) [17], or more [18].

The shallow breathing and periodic pauses lead to a low frequency hypoxia. Thus, the oxygen level decreases and may cause the upper airway muscles to strengthen their tone. Consequently, the upper airway narrows and collapses. As a result, the oxygen supply is not sufficient enough and intermittent hypoxia takes place. This leads to an increase in heart rate, blood pressure, and sympathetic nervous system activity (SNA) which can all lead to heart failure [19]. Previous studies have implicated that OSA may have a connection to SIDS [20] with further reported anatomical changes in SIDS cases that are similar to those in OSA [21]. Kattwinkel et al. [4] states that SIDS cases may reflect a delayed development of arousal or cardiorespiratory control. As OSA causes hypercapnia and hypoxia, it may give grounds to suggest that an abnormal response of infants to these factors may result in SIDS.

This study was performed to test a possible association between SIDS and 24 gene variants that were previously shown to be related to obstructive sleep apnea (OSA) as we hypothesize that both OSA and SIDS might (at least in part) share a common etiology.

## Methods

The SIDS sample group was composed of 282 Caucasoid infants (180 males and 102 females) from Lower Saxony, Germany. Their death occurred within their first year of life and a postmortem examination failed to provide a clear cause of death. The average age at death was 130 days. Data of gender, age, sleeping position, date of birth, and death were gathered from the corresponding autopsy case reports. However, due to anonymization, these data were not available for a small number of cases, that were included into the study nevertheless (exact information on these subgroups and the number of samples included is given in the Data analysis section). The control group consisted of 374 samples (207 males and 167 females). The group included 348 healthy persons, as well as 26 deceased infants (11 males and 15 females) for who the cause of death (other than SIDS) was established after an autopsy. Due to anonymization, the age of the controls was not available. The Hannover Medical School ethics committee has approved the study.

For DNA extraction of the cases and controls, blood samples were used, usually blood which was withdrawn from the heart. We used the QIAamp® DNA Mini Kit (Qiagen, Hilden, Germany) method following the manufacturer’s instructions.

Genotyping was performed on an allele-specific SNPtype Assay which was applied on a 192.24 Dynamic Array and run through a Biomark EP1 platform (Fluidigm Corp., South San Francisco, CA, USA) as described [22]. Fluidigm designed the primers (FAM and HEX-labeled, detecting either one allele of the respective SNP). Primer and probe sequences are given in Tables 1 and 2. A 2× Multiplex PCR Master Mix (Qiagen, Hilden, Germany), 10× SNPtype STA Primer Pool, PCR-certified water, and genomic DNA were combined to produce a specific target amplification (STA) for all 190 samples and two non-template controls (NTCs) per array. The STA was placed into a PCR-System with thermocycling conditions as instructed (15 min at 95 °C followed by 14 cycles of 15 s at 95 °C, and 4 min at 60 °C). Following the PCR, the STA products were diluted 100-fold with 1× TE buffer.

**Table 1:** SNPs with corresponding alleles and genes as well as primer and probe sequences. *ASP*, allele-specific primer allele; *LSP*, locus-specific primer; *STA*, specific target amplification

SNP	Gene	ASP1	ASP2	ASP1	ASP2	LSP	**STA**
rs10160548	*HTR3A*	G	T	TGTGTCCCATCATCACAGGG	CTGTGTCCCATCATCACAGGT	GTACCCAGAGCCTGCTGGA	CCTTAATTGCTGCCCACCT
rs1042714	*ADRB2*	C	G	CCCACACCTCGTCCCTTTG	CCCACACCTCGTCCCTTTC	GAAGCCATGCGCCGGAC	GACGATGCCCATGCCC
rs10515807	*ADRA1B*	C	T	AGGAAAAGCCTAGGAGAGCAC	GAGGAAAAGCCTAGGAGAGCAT	GAGGCCAAAGCTTCCCACAC	AGAGTCAGCTTCAAAATCACACAG
rs10980705	*LPAR1*	C	T	GTAGCTCATTTGGAACATAATGAGCTAG	AGTAGCTCATTTGGAACATAATGAGCTAA	ACAGAATTCAGTTCAGCATCTAGATTAATCCAT	TGCACTGGTACCATTATTCCATTTT
rs11126184o	*PLEK*	A	C	GGTCTGAGTGAAAGCTAGGACA	GGTCTGAGTGAAAGCTAGGACC	CCCCATCTTCCCAGCTCAGG	GCCAAATGAAGGATGACTCCTAG
rs11126184u	*PLEK*	A	C	GGTCTGAGTGAAAGCTAGGACA	GGTCTGAGTGAAAGCTAGGACC	CCCCATCTTCCCAGCTCAGG	GCCAAATGAAGGATGACTCCTAG
rs11763517	*LEP*	C	T	CTTAGGTATTAGAGGGTGGCCATTAC	CTTAGGTATTAGAGGGTGGCCATTAT	CCAGATTAACTGTGGTCATAGTCACTCT	GGGCTTGTAAAACTGTTTTTCCAC
rs1409986	*ANGPT2*	C	T	ACCTTGAAGGATCAATCACAGTAGG	GAACCTTGAAGGATCAATCACAGTAGA	CTTCAGGCTCCTCTTCTTCCCA	CCAAGAACCAACGGAAGGG
rs1799983	*NOS3*	G	T	TGCAGGCCCCAGATGAG	CTGCAGGCCCCAGATGAT	GCACCTCAAGGACCAGCTCG	GTGCTGCCCCTGCTG
rs1800541	*EDN1*	G	T	CAGAATTTTTGTTTGTTCTCCACCAC	CCAGAATTTTTGTTTGTTCTCCACCAA	GTCTTACTGGGCCACTGTGAG	CAGGTTAGACAACTGAGCACC
rs1800629	*TNFA*	A	G	GGCTGAACCCCGTCCT	GGCTGAACCCCGTCCC	GTCCCCAAAAGAAATGGAGGCA	TTTGTGTGTAGGACCCTGGA
rs1801253	*ADRB1*	C	G	CGCAAGGCCTTCCAGC	CCGCAAGGCCTTCCAGG	GCGCGCGCAGCAGAG	CCTTCAACCCCATCATCTACTG
rs2071746	*HIF1A*	A	T	AGCGCTGCTCAGAGCAAT	AGCGCTGCTCAGAGCAAA	AGTTCCTGATGTTGCCCACCA	CGTCCCAGAAGGTTCCAGAAA
rs2337980v2	*CHRNA7*	C	T	TCAAAAAAACACAGGCAGCCAG	TCAAAAAAACACAGGCAGCCAA	GCTTTACTCTGGGGTGCTGGTA	CACAGCCCTACTGGTAAAGAAAA
rs261332	*LIPC*	A	G	CTAACACTTTTTAAAATGATAATAAACCCTTGCATA	AACACTTTTTAAAATGATAATAAACCCTTGCATG	ACTTATTTGGAAAATACAAGTTATTTTTCATAAAATTACA	ACACTTTTTAAAATGATAATAAACCCTTGCA
rs35329661	*ARRB1*	A	G	AGGTCATCCCAAACACTAAAGGATT	AGGTCATCCCAAACACTAAAGGATC	ATGCCCTCCAGTGTCTTCTGAAA	GGGAAGAAGTCTGCAGGAAA
rs472112	*ARRB1*	A	G	ATGTAAGAACACCTGCAGGAAGT	TGTAAGAACACCTGCAGGAAGC	CATGGTGACCAAAGGCTCCTC	GGGAAAAGGTATAAGGAATCGCA
rs5335	*EDNRA*	C	G	GATCAGAGAAGAGATTCCCGGAG	GATCAGAGAAGAGATTCCCGGAC	GCACTCCTCGGTACTCCCAT	AGCATTTCTTCTTGGGTGTGG
rs6295	*HTR1A*	C	G	AAGAAGACCGAGTGTGTCTTCG	AAGAAGACCGAGTGTGTCTTCC	CAATGGCGCGAGAACGGA	GGTCAGTCTCCCAATTATTGCTAA
rs6296	*HTR1B*	C	G	GACTCGCACTTTGACTTGGTTG	GACTCGCACTTTGACTTGGTTC	CGTGCCCAGCGAATCCG	CTTCTTTTCCAGCAGGGCG
rs662799	*APOA5*	A	G	CCAGGAACTGGAGCGAAAGTA	CAGGAACTGGAGCGAAAGTG	CCTGCGAGTGGAGTTCAGC	GGCCAGGGACTCTGAGC
rs7030789	*LPAR1*	A	G	TCACTTGACGGTATTATGGTAGTCTACT	CACTTGACGGTATTATGGTAGTCTACC	ACTGTGGAAAGTGAAGCTTCGGA	TTGGACGGGGTGCTATCT
rs769449	*APOE*	A	G	CTCCTGGCCCCATTCAGA	TCCTGGCCCCATTCAGG	GGAAGCAGCACAGAAGCCTC	CCTCTCATCCTCACCTCAACC
rs977214	*PTGER3*	A	G	GACATTGGTAGTATGGTCTCTCATTTCT	ACATTGGTAGTATGGTCTCTCATTTCC	GCAGATCTCTGGATACGTTCCAGT	GCTTCTTTGCTCTCATCTTAAAGACA

**Table 2:** Selected association results with all associations at *p* < 0.05

Stratum	Gene	SNP	Obs	Case/controls	OR (95% CI)	**p value**
Overall						
	*ADRB2*	rs1042714	653	281/372	0.79 (0.64; 0.99)	0.04
	*EDN1*	rs1800541	656	282/374	1.34 (1.02; 1.84)	0.038
Subgroups						
Male	*ADRB2*	rs1042714	386	180/206	0.74 (0.55; 0.99)	0.04
Female	*ADRA1B*	rs10515807	269	102/167	1.63 (1.02; 2.63)	0.043
2-4 months	*ADRB2*	rs1042714	462	90/372	0.71 (0.51; 1.00)	0.05
2-4 months	*ARRB1*	rs35329661	465	91/374	2.58 (1.11; 5.96)	0.027
2-4 months	*ARRB1*	rs472112	464	91/373	0.70 (0.50; 0.97)	0.034
4-6 months	*LPAR1*	rs10980705	411	38/373	0.50 (0.26; 0.98)	0.044
4-6 months	*EDN1*	rs1800541	412	38/374	2.01 (1.12; 3.59)	0.019
8-10 months	*ADRA1B*	rs10515807	399	25/374	2.12 (1.08; 4.18)	0.029
Spring	*ARRB1*	rs472112	435	62/373	0.65 (0.44; 0.96)	0.032
Spring	*LPAR1*	rs7030789	435	62/373	1.77 (1.18; 2.65)	0.006
Summer	*ADRB1*	rs1801253	313	66/247	1.53 (1.03; 2.29)	0.037
Summer	*ARRB1*	rs472112	439	66/373	0.68 (0.46; 0.99)	0.043
Summer	*HTR1A*	rs6295	440	66/374	1.53 (1.06; 2.21)	0.023
Autmn	*TNFA*	rs1800629	436	62/374	0.03 (1.05; 2.66)	0.031
Winter	*EDNRA*	rs5335	435	64/371	0.69 (0.48; 1.00)	0.049
Not in prone position	*LIPC*	rs261332	585	216/369	0.67 (0.49; 0.93)	0.015
Prone position	*ADRB2*	rs1042714	433	61/372	0.62 (0.42; 0.94)	0.022
Prone position	*ADRA1B*	rs10515807	436	62/374	1.86 (1.17; 2.94)	0.008
Prone position	*EDNRA*	rs5335	432	61/371	0.61 (0,42; 0.89)	0.01
Prone position	*HTR1A*	rs6295	436	62/374	1.46 (1.01; 2.11)	0.046
Obs observation						

*OR*, odds ratio; *95% CI* 95%, confidence interval

A sample mix and a 10× assay mix must be prepared prior to loading the biochip. The 10× Assay Mix consists of 2 μl 2× Assay Loading Reagent (Fluidigm), 1.2 μl PCR-certified water, and 1 μl SNPtype assay mix (Fluidigm). The sample mix is comprised of 2.25 μl 2× Fast Probe Master Mix (Biotium, Hayward, CA, USA), 0.225 μl20× SNP Type Sample Loading Reagent (Fluidigm), 0.075 μl SNP Type Reagent (Fluidigm), 0.027 μl ROX (Invitrogen, Carlsbad, CA, USA), 0.048 μl PCR-certified water, and 2 μl STA product (1:100). As instructed, 3 μl of each 10× Assay Mix and sample mix were pipetted into the separate inlets of the Dynamic Array. In total, 190 samples were placed on each array, two NTCs and 24 SNPtype assays. Samples and assays were mixed using the IFC Controller RX (Fluidigm). Next, the Dynamic Array was set into the Biomark HD System (Fluidigm) for thermocycling following the default PCR protocol. After each PCR cycle, FAM and HEX signals were identified and genotype calls were received. The resulting data was analyzed by the Fluidigm SNP Genotyping Analysis software [20]. Seventy-six samples were run in duplicates for internal quality control. Given in Table 1 are the sequences of the primers and probes used as well as the genes and SNPs tested.

### Selection of loci

In this study, candidate genes were selected that were thought to be associated with the development of OSA which is involved in many body processes. Information on candidate loci was retrieved from various publications [15, 16, 23–27]. Using these sources, 49 SNPs in 35 genes were considered. Then SNPs that were already looked at in different studies (for example rs6311 or rs1042173 [20]) were ruled out. Additionally, in cases that two SNPs were lying on the same gene and were linked, meaning they are always inherited together, the SNP with the higher frequency of the minor allele was used. Furthermore, those with a low frequency (MAF ≤ 0.063) of the minor allele were omitted based on power calculations. Lastly, SNPs were excluded in genes that seemed not to be related to the response of stress, oxygen, or hypoxia. With this procedure, 24 SNPs in 21 genes were found and used that had published evidence for an association with OSA and its symptoms.

### Data analysis

Two different researchers manually inspected the cluster plots, and the Hardy-Weinberg equilibrium (HWE) was tested using *χ*^2^ tests. One single-nucleotide polymorphism (SNP) with significant deviation from HWE was omitted, leaving 23 loci for further analysis.

Statistical analysis was performed using univariate logistic regression analyses with STATA v.12.0. The main analysis compared all SIDS cases (*n* = 282) vs controls (*n* = 374). The four subcategories were compared within the SIDS samples which were known to increase the risks of SIDS that have been previously demonstrated [16, 17]. These were (1) gender (males = 180; females = 102), (2) age group (6 test models: 0–2 months (*n* = 63), 2–4 months (*n* = 91), 4–6 months (*n* = 38), 6–8 months (*n* = 23), 8–10 months *(n* = 25), 10–12 months (*n* = 17)), (3) time of death by season as a proxy for temperature (4 test models: spring (*n* = 62), summer (*n* = 66), autumn (*n* = 62), winter (*n* = 65), and (4) sleeping in a prone position (*n* = 62) compared to not sleeping in a prone position or unknown (*n* = 220). After this stratification, logistic regression analyses were repeated with the respective subset of SIDS cases compared to all controls. Two-sided *p* values were considered noteworthy if *p* < 0.05 and significant if *p* < 0.00015 (Bonferroni correction for 336 tests).

## Results

In this study, a total of 656 samples (SIDS = 282, controls = 374) were successfully genotyped for 23 SNPs, with a call rate of 95% and a concordance rate of 99.5% in 76 duplicates. Twelve SNPs showed a nominally significant *p* value of equal or less than 0.05 in any of the five categories. These are summarized in Table 2. Eight of them were located in or near cardiorespiratory genes and were associated in almost all categories.

In the main analysis of all cases and controls, the two SNPs rs1042714 (*p* = 0.040; OR = 0.79; 95% CI 0.64; 0.99) and rs1800541 (*p* = 0.038; OR = 1.37; 95% CI 1.02; 1.84)), which are both located in cardiovascular genes, *ADRB2* and *EDN1* respectively, showed evidence for an overall association at *p* < 0.05. Moreover, these SNPs had significant values in one or more of the other subcategories. The SNP rs1042714 in *ADRB2* proves a relevant *p* value in three other subgroups: “males only,” “2–4 months,” and “prone sleep position.” The subgroup “4–6 months” was additionally significant in the SNP rs1800541.

Two other SNPs gave significant results at *p* < 0.05 in three subgroups: SNP rs10515807 in *ADRA1B* (subgroups “females only”, “8–10 months,” and “prone sleep position”) and SNP rs472112 in *ARRB1* (subgroups “2–4 months,” “spring,” and “summer”). Four SNPs were associated with sleeping in a prone position (rs1042714, rs10515807, rs5335 (all in *EDNRA*) and rs6295 in *HTR1A* which has been suggested to be a huge risk factor for SIDS [2]. All of these SNPs are located in cardiorespiratory genes.

The other four non-cardiorespiratory SNPs (rs261332, rs1800629, rs10980705, and rs7030789) that had a significant value on *p* < 0.05 were lying on three different genes (*LIPC*, *TNFA*, and *LPAR1*) respectively. However, each of them was significant in only one subcategory. Rs261332 in *LIPC* showed evidence in “not lying in a prone position” (*p* = 0.015; OR 0.67; 95% CI 0.49; 0.93). *TNFA* (rs1800629) has a significant value in the category “autumn” (*p* = 0.031; OR = 1.67; 95% CI 1.05; 2.66). *LPAR1*, that encodes lysophosphatidic acid receptor 1, has two SNPs lying on it, which are independently inherited, show significant values at the subcategories “4–6 months” (rs10980705; *p* = 0.044; OR = 0.50; 95% CI 0.26; 0.98) and “spring” (rs7030789; *p* = 0.006; OR = 1.77; 95% CI 1.18; 2,65). The 10 SNPs for that a significant association could be demonstrated were searched in the GTEx Portal (https://gtexportal.org/home/) in order to extract information on the influence this variant exerts on the gene expression.

Accordingly, the minor alleles for the SNPs rs1800541,rs1042714, rs10980705, rs1801253, rs6253, rs1800629, rs5335, and rs261332 were associated with a weaker expression of the gene in at least one tissue. For the remaining SNPs no information was available.

## Discussion

We hypothesized that both OSA and SIDS might share (at least in part) a common etiology. Among the similarities are, e.g., the involvement of the (cardio) respiratory system or the increased prevalence in males [2]. If this should be true, gene variants already associated with OSA should also be associated with SIDS. In fact, we found evidence for such an association for several loci.

In total, 12 of the selected 24 SNPs showed evidence for an association at *p* < 0.05 with SIDS in any of the groups. Eight of these SNPs were located in or near one of seven cardiorespiratory genes. The two SNPs that were nominally significant in the main analysis were located at the gene loci for *ADRB2* and *EDN1*, respectively. *ADRB2* is an adrenergic receptor that is active in the cardiovascular as well as in the respiratory system. On the heart, activation of this receptor has a positive chrono- and ionotropic effect [28]. In the bronchi, it usually facilitates relaxation of the bronchi as a result of smooth muscle relaxation [29, 30]. *EDN1* encodes endothelin-1 and is activated by hypoxia [31]. When stimulated, it leads to vasoconstriction [32, 33] and over time it can generate pulmonary arterial hypertension since it also causes a positive iono- and chronotropic effect on the heart [33]. Consequently, it is known to play a role in heart failure [34, 35]. Additionally, through hormones, it can modulate the cardiorespiratory center. Both gene candidates are important factors in OSA as they affect the respiratory and cardiac systems.

We found that four SNPs at cardiorespiratory genes (*ADRB2*, *ADRA1B*, *HTR1A*, and *EDNRA*) showed evidence of association in the subgroup “prone sleeping position.” This supports the assumption that prone sleeping promotes heart and breathing problems in SIDS and that abnormalities in the noradrenergic and/or the serotonergic system, potentially in combination with the Endothelin receptor type A increase the deleterious effect of a prone position. These findings also support previous evidence that these genes constitute strong risk factors for SIDS. As mentioned before, *ADRB2* causes stress on the heart, as well as *ADRA1B*. *ADRB2* variant rs1042714 is also significant in the subcategory “males only” which is coherent with the well-known gender bias to male infants. *EDNRA* encodes the receptor for endothelin-1, which is activated by hypoxia. *HTR1A* on the other hand, encodes a serotonin receptor (1a) that has always been in close focus in many SIDS investigations. In 75% of SIDS cases, a decreased expression of *HTR1A* was found. Moreover, male SIDS cases have additionally demonstrated a greater reduction in Serotonin receptor 1a than female cases [36], which is consistent with the incidence rate of SIDS. Hence, there seems to be a noteworthy association between the serotonin-pathway and SIDS even though, in another study a different SNP, which also lies in the *HTR1A gene* did not show any significance [20]. *HTR1A* is key for autonomic responses to cardiorespiratory regulation and homeostatic stress [37]. Stimulation of the receptor in the raphe nuclei causes a decrease in ventilatory response to hypercapnia, fragmented sleep with reduced body temperature, heart rate and body movement, and a reduction in cardiovascular response to stress [20–22, 38]. In a different study [39], knock-out mice were produced with an overstimulation of serotonin. Most of these mice did not reach the age of 3 months. Moreover, they had sporadic autonomic crisis, which expressed in severe bradycardia and hypothermia that also progressed to death. These mice share critical features with SIDS cases as they also revealed a pronounced bradycardia that proceeded apnea [40]. Sleeping in a prone position has been associated with altered autonomic control, manifested by raised heart rates [23–30, 41], decreased heart rate variability [25, 30–34], and increased sympathetic tone[25, 26, 31, 32, 35, 36] which can cause heart failure. Hunt et al. [42] stated that in autopsy findings, there were more often pulmonary congestion and edema in SIDS cases than in other infants which indicates terminal left ventricular failure.

Both OSA and SIDS are primarily respiratory conditions, but in both the cardiovascular system is of importance as well. OSA causes an increase in heart rate and blood pressure over night which leads to arousal. This process “back to sleep–repeat” can happen up to 100 times a night. As a result, there is no resting phase and the cardiovascular system is exposed to stress. Thus, intermittent hypoxia, oxidative stress, systemic inflammation, exaggerated negative thoracic pressure, sympathetic over-activation, and increase in blood pressure [19] are problems that further strain on the circulation system. The apnea causes an elevation in the left ventricular transmural pressure (afterload), as a result of hypoxia, arousal of sleep, and increased sympathetic nervous system activity. that is also further amplified due to the suppression of the sympathetic inhibitory effects of lung stretch receptors by apnea. These factors cause great stress on the heart and predispose a patient to cardiac ischemia, arrhythmias, and heart failure [17]. This correlation between respiration and circulation might be a major impact on SIDS as well, for which a cardiovascular role is suspected for years [43, 44].

The other four non-cardiorespiratory SNPs (rs261332, rs1800629, rs10980705, rs7030789) that had a nominally significant value on *p* < 0.05 were lying on three different genes (*LIPC*, *TNFA*, *and LPAR1*) respectively. *TNFA* (rs1800629) encodes for the tumor necrosis factor alpha. This cytokine, is one of several that have been described to be increased in OSA [45] and hence has shown an association to it [15]. However, the mechanism of action is not well understood. Interestingly, tumor necrosis factor alpha and polymorphisms in the *TNFA* gene as well as other cytokins have been investigated and linked to SIDS in many studies [22, 46–49]. It is possible that this pro-inflammatory cytokine could affect the respiratory network, which would be consistent with the fact that OSA also is correlated to *TNFA*.

*LPAR1*, that encodes lysophosphatidic acid receptor 1, has been shown to have a connection to OSA as well [23]. However, this is the first evidence for a possible link to SIDS. As these associations discussed above were only found in subgroups and do not withstand correction for multiple testing, they need to be confirmed in additional studies.

## Conclusion

This study is to our knowledge the first to look at susceptibility genes involved in obstructive sleep apnea, a respiratory disease also associated with heart problems, while searching for a connection to SIDS. It represents a new set of data that has not previously been published. The evidence found in this study corroborates the hypothesis of a correlation between SIDS and genes related to OSA and hence the cardiovascular system. However, further replication testing should take place with a larger sample group.

## Supplementary Information

Table S1: Concise information on the function of the genes and gene variants typed herein (DOCX 33 kb)

Table S2: Allelic frequencies for the 24 SNPs typed herein in cases and controls (XLSX 11 kb)

## Abbreviations

SIDS: Sudden infant death syndrome
OSA: Obstructive sleep apnea
SNPs: Single-nucleotide polymorphisms
CI: Confidence interval
SNA: Sympathetic nervous system activity
