# Supplementary material for: Gene variants associated with obstructive sleep apnea (OSA) in relation to sudden infant death syndrome (SIDS)
Source: Int J Legal Med. 2021 Feb 8;135(4):1499–506. doi: 10.1007/s00414-020-02480-0 (PMC8206047; doi:10.1007/s00414-020-02480-0)
Supplement: Supplementary file 1 — Concise information on the function of the genes and gene variants typed herein (DOCX 33 kb) [file 414_2020_2480_MOESM1_ESM.docx]

Explanation of the function of the Obstructive Sleep Apnea (OSA) related SNPs

SNP – single nucleotide polymorphism

OSA – obstructive sleep apnea

O2 – oxygen

HDL – high density lipoprotein

| SNP | Gene | Function |
| --- | --- | --- |
| rs10160548 | HTR3A - 5-hydroxytryptamine  (serotonin) receptor 3A | - G-coupled signaling pathway - causes fast, depolarizing responses in neurons after activation activated by serotonin, hormone and mitogen - response to O2 compounds |
| rs1042714 | ADRB2 - Adrenoceptor beta 2,  surface | - G-coupled signaling pathway - regulates blood vessel size - regulates heart contraction - regulates blood coagulation - response to O2 compounds - response to O2 levels - response to gas exchange - response to hypoxia - susceptibility to asthma |
| rs10515807 | ADRA1B - Adrenoceptor alpha 1B | - G-coupled signaling pathway - regulation of dopaminergic neurotransmission - regulates heart contraction - response to O2 compounds |
| rs10980705 | LPAR1 - Lysophosphatidic acid  receptor 1 | - G-coupled signaling pathway - response to O2 levels |
| rs11126184o | PLEK - Pleckstrin | - G-coupled signaling pathway - reg. blood coagulation - response to stress |
| rs11126184u | PLEK - Pleckstrin | - G-coupled signaling pathway - reg. blood coagulation - response to stress |
| rs11763517 | LEP - Leptin | - involved in the regulation of immune and inflammatory responses, hematopoiesis, angiogenesis and wound healing - influence functional outcome of lung as it is induced by alveolar interstitial fibroblasts - regulates vasoconstriction - response to stress - response to O2 compounds - response to O2 levels - response to hypoxia |
| rs1409986 | ANGPT2 - Angiopoietin-2 | - expressed only at the sites of vascular remodelling - response to O2 compounds - response to O2 levels - response to hypoxia |
| rs1799983 | NOS3 - Nitric oxide synthase 3  (endothelial cell) | - reactive free radical - enzyme which is responsible for the production nitric oxide - regulates blood vessel size - regulates heart contraction - blood circulation - regulates blood coagulation - response to stress - response to O2 compounds |
| rs1800541 | EDN1 - Endothelin 1 | - G-coupled signaling pathway - regulates blood vessel size - vasoconstriction - regulates vasoconstriction - blood circulation - artery smooth muscle contraction - regulates blood coagulation - response to stress - response to O2 compounds - response to O2 levels - response to gas exchange - activated by hypoxia |
| rs1800629 | TNFA - Tumour necrosis factor  alpha | - mainly secreted by macrophages - proinflammatory cytokine - response to oxidative stress |
| rs1801253 | ADRB1 - Adrenoceptor beta 1 | - increases heart rate - G-coupled signaling pathway - regulates blood vessel size - regulates heart contraction - regulates vasoconstriction - response to stress |
| rs2071746 | HIF1A - Hypoxia inducible factor  1, alpha subunit | - regulator of cellular and systemic homeostatic response to hypoxia - response to stress - embryonic vascularisation (angiogenesis) |
| rs2337980v2 | CHRNA7 - α7-acetylcholine  receptor | - response to stress - response to O2 levels - response to hypoxia |
| rs261332 | LIPC - Lipase, hepatic | - response to oxidative stress - increase HDL - biomarker for cardiovascular diseases |
| rs35329661 | ARRB1 - Arrestin beta 1 | - G-coupled signaling pathway - response to stress - protein that can stabilize HIF-1α under hypoxic conditions |
| rs472112 | ARRB1 - Arrestin beta 1 | - G-coupled signaling pathway - response to stress - protein that can stabilize HIF-1α under hypoxic conditions |
| rs5335 | EDNRA - Endothelin receptor  type A | - G-coupled signaling pathway - regulates blood vessel size - causes vasoconstriction - artery smooth muscle contraction - increases blood pressure - response to stress - response to O2 levels - response to gas exchange - response to hypoxia |
| rs6295 | HTR1A - 5-hydroxytryptamine  (serotonin) receptor 1A | - G-coupled signaling pathway - regulates blood vessel size - vasodilation - stimulates Nervus Vagus - decreases heart rate - decreases blood pressure - response to stress |
| rs6296 | HTR1B - 5-hydroxytryptamine  (serotonin) receptor 1B | - regulates blood vessel size - vasoconstriction - response to stress - response to O2 compounds |
| rs662799 | APOA5 - Apolipoprotein A-V | - response to stress |
| rs7030789 | LPAR1 - Lysophosphatidic acid  receptor 1 | - G-coupled signaling pathway - response to O2 levels |
| rs769449 | APOE - Apolipoprotein E | - apoprotein of the chylomicron - essential for the normal catabolism of triglyceride-rich lipoprotein constituents - could predispose to OSA - by lowering levels of choline actyltransferase and reducing neuromuscular activation of upper airway dilator muscles - G-coupled signaling pathway - regulates blood vessel size - vasoconstriction - blood circulation - regulates blood coagulation - response to stress - response to O2 compounds |
| rs977214 | PTGER3 - prostaglandin EP3  receptor | - response to stress - response to O2 compounds |
